# Reactivation of hepatitis B (reverse seroconversion) after melphalan/dexamethasone therapy for primary amyloidosis: a case report

**DOI:** 10.1186/s13256-015-0610-z

**Published:** 2015-06-02

**Authors:** Woo-Ram Moon, Do-Sik Moon, JoA Kim, Young-min Yoon, Byung-Seok Choi, Choon-Hae Chung, Sang-Gon Park

**Affiliations:** 1 Department of Internal Medicine, Chosun University Hospital, 365 Pilmun-daero, Dong-gu Gwangju, 501-717 Republic of Korea; 2 Department of Internal Medicine, Hemato-oncology, Chosun University Hospital, 365 Pilmun-daero, Dong-gu Gwangju, 501-717 Republic of Korea; 3 Department of Medicine, Graduate School of Chosun University, 309 Pilmun-daero, Dong-gu Gwangju, 501-717 Republic of Korea

**Keywords:** Hepatitis B, Immunity, Innate, Amyloidosis, Dexamethasone, Melphalan

## Abstract

**Introduction:**

Hepatitis B virus (HBV) reactivation (so-called reverse seroconversion) is a rare but known complication of hematopoietic stem cell transplantation, immunosuppressive therapy, or high-dose chemotherapy plus rituximab. This event is linked to a treatment-related fall in titers of antibodies to hepatitis B surface antigen (HBsAb) below the protective threshold level.

**Case presentation:**

A 77-year-old Korean man diagnosed with primary amyloidosis was started on melphalan/dexamethasone combination therapy. During treatment, laboratory indices of hepatic function suddenly deteriorated, and he developed acute hepatitis through reverse HBV seroconversion, becoming positive for hepatitis B surface antigen (HBsAg) and negative for HBsAb. HBV DNA was also detectable in serum to a profound extent. Normal liver function was gradually restored during the course of antiviral therapy (entecavir).

**Conclusions:**

HBV reactivation may lead to fatal liver disease in a significant percentage of patients. As a result, physicians often screen for HBsAg and HBsAb prior to initiating chemotherapy, advising antiviral treatment in patients seropositive for HBsAg, even in the absence of hepatitis B e antigen. Here, a case of HBV reactivation is described, involving a patient given relatively low-dose chemotherapy (melphalan/dexamethasone) for primary amyloidosis.

## Introduction

Instances of hepatitis B virus (HBV) reactivation (so-called reverse seroconversion) have been reported after hematopoietic stem cell transplantation or high-dose chemotherapy plus rituximab and are seldom-observed consequences of multiple myeloma. However, there are no known published accounts of HBV reactivation following melphalan/dexamethasone treatment of primary amyloidosis [1-5].

## Case presentation

A 77-year-old Korean man presented to our hospital with an axillary mass. The presence of amyloid in the subsequent excisional biopsy prompted a bone marrow biopsy, related laboratory tests, and pertinent imaging studies, including computed tomography (CT). Ultimately, a diagnosis of primary amyloidosis was rendered.

Despite the patient’s advanced age, melphalan/dexamethasone combination therapy was elected. After three cycles of this regimen, detectable masses grew smaller and his serum kappa/lambda ratio normalized, so treatment continued. However, serum levels of aspartate aminotransferase (AST) and alanine aminotransferase (ALT) rose precipitously after six cycles of therapy.

Our patient denied use of other medications (including herbal or dietary supplements), smoking, or consumption of alcohol, and there was no history of blood transfusion. On physical examination, he was alert, with normal blood pressure, pulse, and respiratory rate. No direct or rebound tenderness of the abdomen was evident.

Laboratory tests yielded the following values: white blood cell count, 2,720/mm^3^ (40.1% neutrophils, 43.5% lymphocytes, 1.4% eosinophils); hematocrit, 10.6g/dL; platelet count, 111,000/mm^3^; prothrombin time, 11.8 seconds; AST level, 454.1IU/L; ALT level, 496.3IU/L; total bilirubin, 1.34mg/dL; direct bilirubin, 1.04mg/dL; albumin, 3.11g/d; alkaline phosphatase (ALP), 142U/L; gamma-glutamyl transferase (rGTP), 126U/L; blood urea nitrogen (BUN), 17.2mg/dL; and creatinine (Cr), 1.28mg/dL.

Prior to initiating chemotherapy, our patient had been screened twice for hepatitis, testing negative for hepatitis B surface antigen (HBsAg, 0.41S/Co) and positive for antibodies to HBsAg (HBsAb, 55.0mIU/mL). No further serologic testing was pursued at this juncture, but after receiving chemotherapy, HBsAg seroconversion (5,592S/Co) and loss of HBsAb (0.62mIU/mL) were documented. Repeat testing generated the same results, so screening was expanded to include hepatitis B envelope antigen (HBeAg, negative (0.27S/Co)), antibodies to HBeAg (HBeAb, positive (0.02S/Co)), and antibodies to hepatitis B core antigen (immunoglobulin M (IgM) HBcAb, negative (0.27S/Co); immunoglobulin M (IgG) HBcAb, positive (2.2S/Co)). A massive HBV DNA burden (67,322,328 copies/mL) was also determined by real-time polymerase chain reaction (PCR).

**Figure 1:**
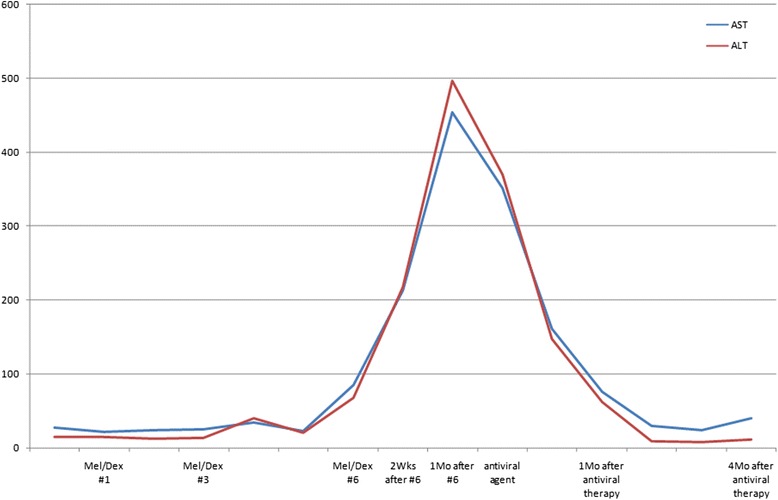
Changes of values of liver function tests and viral indicators.

Given a lack of HBV vaccination by history, the patient’s baseline HBsAb positivity was attributed to innate (naturally acquired) immunity. Hence, this acute bout of hepatitis was considered HBV reactivation. Entecavir antiviral treatment was then administered, gradually restoring normal liver function within three weeks. Five months later, AST and ALT levels were 40IU/L and 12IU/L, respectively. At this point, he also tested negative for HBsAg and positive for HBsAb, with PCR-determined HBV DNA level at 3,600 copies/mL (Figure 1, Table 1). Continued chemotherapy for primary amyloidosis was declined, and he died unexpectedly seven months later.

**Table 1:** **Values of viral indicators**

	**HBsAg (S/Co)**	**HBsAb (mIU/mL)**	**HBeAg (S/Co)**	**HBeAb (mIU/mL)**	**IgM-HBcAb (S/Co)**	**IgG-HBcAb (S/Co)**	**HBV DNA PCR (copies/mL)**
**Before CTx**	(—) 0.40	(+) 85.2					
	(—) 0.41	(+) 55.0					
**Mel/Dex#6**	(+) 5,592	(—) 0.62					
	(+) 5,667	(—) 0.09	(—) 0.27	(+) 0.02	(—) 0.27	(+) 12.2	67,422,428
**After antiviral agent**							3,600

CTx, chemotherapy; Dex, dexamethasone; HBcAb, hepatitis B core antigen; HBeAb, antibodies to HBeAg; HBeAg, hepatitis B envelope antigen; HBsAb, antibodies to HBsAg; HBsAg, hepatitis B surface antigen; HBV, hepatitis B virus; Mel, melphalan; PCR, polymerase chain reaction.

## Discussion

HBV infection remains a major cause of acute and chronic liver disease. According to the World Health Organization (WHO), 350 to 400 million people worldwide suffer from chronic HBV infection [6], with relatively greater prevalence in Korea (2 to 10%) [7]. Presence of HBsAb and HBcAb is equated with resolution of infection and HBV clearance. In addition to enabling viral clearance during acute infection, immune reactivity to HBV antigens is also responsible for disease pathogenesis [8].

Innate immunity is important in controlling HBV infection immediately after onset, generally limiting the spread of infection and instigating an efficient adaptive immune response. Organism-specific structures (for example, double-stranded RNA and bacterial wall components) of pathogens trigger pattern-recognition receptors (PRRs) to activate innate immunity. The most important PRRs in viral infections are Toll-like receptors (TLRs), RNA helicases (such as retinoic acid-inducible gene I (RIG-I) and melanoma differentiation-associated gene 5 (MDA5)), and double-stranded RNA-dependent protein kinase (PKR) [8,9].

Adaptive immunity is responsible for immunity to HBV infection conferred by vaccine administration. Routine HBV vaccination at birth is recommended for a number of reasons. Approximately 90% of infants born to HBV-infected mothers will acquire the infection at birth; and 30 to 50% of infected youths <5 years of age become chronic HBV carriers, compared with only 6 to 10% of newly infected adults [8-10]. The series of immunizations constituting recombinant HBV vaccines are 95% effective at inducing seroimmunity. Although in use for only 20 years, their duration is likely lifelong [11,12].

Reverse seroconversion of HBV due to medical treatments, such as hematopoietic stem cell transplantation and high-dose chemotherapy plus rituximab, is an uncommon event that seldom occurs with multiple myeloma as well. During chemotherapy, amplified viral replication increases DNA polymerase activity, thereby boosting serum levels of HBV DNA, HBeAb, and HBsAg; reversing HBsAb titers; and enabling the spread of liver infection [2]. The immunologic reconstruction that follows cytotoxic treatments is marked by massive and rapid immune-mediated destruction of infected hepatocytes, reflected clinically in rising AST and ALT levels. Such scenarios may culminate in serious and even fatal liver disease for a significant percentage of patients [1-5].

Prior to initiating any cytotoxic or immunosuppressive therapy, patients should be adequately screened for HBV infection. Antiviral treatment is advised for HBsAg-positive states (even if negative for HbeAg) [10,13].

As seen here, HBV reactivation may result from relatively low-dose chemotherapy (melphalan/dexamethasone), calling for expanded testing in similar circumstances.

## Conclusions

This patient (HBsAg-negative/HBsAb-positive status at baseline) illustrates that HBV reactivation (that is reverse HBV seroconversion) may develop as an exceedingly rare consequence of melphalan/dexamethasone therapy for primary amyloidosis. Comprehensive HBV screening, including HBcAg, HBcAb, and HBV DNA serologic testing, is clearly needed in this setting.

## Consent

Written informed consent was obtained from the patient and the patient’s legal guardians for publication of this case report and any accompanying images. A copy of the written consent is available for review by the Editor-in-Chief of this journal.

## Abbreviations

ALP: alkaline phosphatase
ALT: alanine aminotransferase
AST: aspartate aminotransferase
BUN: blood urea nitrogen
Cr: creatinine
CT: computed tomography
HBcAb: hepatitis B core antigen
HBeAb: antibodies to HBeAg
HBeAg: hepatitis B envelope antigen
HBsAb: antibodies to HBsAg
HBsAg: hepatitis B surface antigen
HBV: hepatitis B virus
IgG: immunoglobulin G
IgM: immunoglobulin M
MDA5: melanoma differentiation-associated gene 5
PKR: RNA-dependent protein kinase
PRRs: pattern-recognition receptors
rGTP: gamma-glutamyl transferase
RIG-I: retinoic acid-inducible gene I
PCR: polymerase chain reaction
TLRs: Toll-like receptors
WHO: World Health Organization

## References

[1] Yoshida T, Kusumoto S, Inagaki A, Mori F, Ito A, Ri M, et al. Reactivation of hepatitis B virus in HBsAg-negative patients with multiple myeloma: two case reports. Int J Hematol. 2010;91:844–9. 10.1007/s12185-010-0592-y 20473594

[2] Lim SM, Jang JW, Kim BW, Choi H, Choi KY, Park SJ, et al. Hepatitis B virus reactivation during chlorambucil and prednisone treatment in an HBsAg-negative and anti-HBs-positive patient with B-cell chronic lymphocytic leukemia. Korean J Hepatol. 2008;14:213–8. 10.3350/kjhep.2008.14.2.213 18617769

[3] Woo SY, Cho SH, Lee SM, Koh MB, Noh CH, Kim CW, et al. Change in the serologic markers of hepatitis B after allogenic hematopoietic stem-cell transplantation. Korean J Hepatol. 2009;15:131–9. 10.3350/kjhep.2009.15.2.131 19581765

[4] Kusumoto S, Tanaka Y, Mizokami M, Ueda R. Reactivation of hepatitis B virus following systemic chemotherapy for malignant lymphoma. Int J Hematol. 2009;90:13–23. 10.1007/s12185-009-0359-5 19544079

[5] Thompson PA, Tam CS, Thursky K, Seymour JF. Hepatitis-B reactivation and rituximab-containing chemotherapy: an increasingly complex clinical challenge. Leuk Lymphoma. 2010;51:1592–5. 10.3109/10428194.2010.509456 20807092

[6] Lok AS, McMahon BJ. Chronic hepatitis B. Hepatology. 2001;34:1225–41. 10.1053/jhep.2001.29401 11732013

[7] Jeong S, Yim HW, Bae SH, Lee WC. Changes of hepatitis B surface antigen seroprevalence in Korea, 1998–2005. Korean J Epidemiol. 2008;30:119–27.

[8] Bertoletti A, Gehring AJ. The immune response during hepatitis B virus infection. J Gen Virol. 2006;87:1439–49. 10.1099/vir.0.81920-0 16690908

[9] Busca A, Kumar A. Nate immune responses in hepatitis B virus (HBV) infection. Virol J. 2014;11:22. 10.1186/1743-422X-11-22 PMC3922976 24507433

[10] Lok AS, McMahon BJ. Chronic hepatitis B: update 2009. Hepatology. 2009;50:661–2. 10.1002/hep.23190 19714720

[11] Mast EE, Weinbaum CM, Fiore AE, Alter MJ, Bell BP, Finelli L, et al. A comprehensive immunization strategy to eliminate transmission of hepatitis B virus infection in the United States: recommendations of the Advisory Committee on Immunization Practices (ACIP) Part II: immunization of adults. MMWR Recomm Rep. 2006;55:1–33. 17159833

[12] Mast EE, Margolis HS, Fiore AE, Brink EW, Goldstein ST, Wang SA, et al. A comprehensive immunization strategy to eliminate transmission of hepatitis B virus infection in the United States: recommendations of the Advisory Committee on Immunization Practices (ACIP) part 1: immunization of infants, children, and adolescents. MMWR Recomm Rep. 2005;54:1–31. 16371945

[13] Tanaka E, Umemura T. History and prevention of de novo hepatitis B virus-related hepatitis in Japan and the world. Clin J Gastroenterol. 2008;1:83–6. 10.1007/s12328-008-0030-3 26193642

